# Pectobacterium araliae sp. nov., a pathogen causing bacterial soft rot of Japanese angelica tree in Japan

**DOI:** 10.1099/ijsem.0.006326

**Published:** 2024-04-16

**Authors:** Hiroyuki Sawada, Nobutaka Someya, Tomohiro Morohoshi, Mitsuaki Ono, Mamoru Satou

**Affiliations:** 1 Research Center of Genetic Resources, National Agriculture and Food Research Organization (NARO), 2-1-2 Kannondai, Tsukuba, Ibaraki 305-8602, Japan; 2 Institute for Plant Protection, NARO, 2-1-18 Kannondai, Tsukuba, Ibaraki 305-8666, Japan; 3 Graduate School of Regional Development and Creativity, Utsunomiya University, 7-1-2 Yoto, Utsunomiya, Tochigi 321-8585, Japan; 4 Yamanashi Agritechnology Center (retired), 1100 Shimoimai, Kai, Yamanashi 400-0105, Japan

**Keywords:** *Aralia elata*, bacterial soft rot disease, *Erwinia carotovora*, Japanese angelica tree, Japanese aralia, *Pectobacterium carotovorum*

## Abstract

Phytopathogenic bacteria (MAFF 302110^T^ and MAFF 302107) were isolated from lesions on Japanese angelica trees affected by bacterial soft rot in Yamanashi Prefecture, Japan. The strains were Gram-reaction-negative, facultatively anaerobic, motile with peritrichous flagella, rod-shaped, and non-spore-forming. The genomic DNA G+C content was 51.1 mol % and the predominant cellular fatty acids included summed feature 3 (C_16 : 1_ ω7*c* and/or C_16 : 1_ ω6*c*), C_16 : 0_, summed feature 8 (C_18 : 1_ ω7*c* and/or C_18 : 1_ ω6*c*), summed feature 2 (comprising any combination of C_12 : 0_ aldehyde, an unknown fatty acid with an equivalent chain length of 10.928, C_16 : 1_ iso I, and C_14 : 0_ 3OH), and C_12 : 0_. Phylogenetic analyses based on 16S rRNA and *gyrB* gene sequences, along with phylogenomic analysis utilizing whole-genome sequences, consistently placed these strains within the genus *Pectobacterium*. However, their phylogenetic positions did not align with any known species within the genus. Comparative studies involving average nucleotide identity and digital DNA–DNA hybridization with the closely related species indicated values below the thresholds employed for the prokaryotic species delineation (95–96 % and 70 %, respectively), with the highest values observed for *Pectobacterium polonicum* DPMP315^T^ (92.10 and 47.1 %, respectively). Phenotypic characteristics, cellular fatty acid composition, and a repertoire of secretion systems could differentiate the strains from their closest relatives. The phenotypic, chemotaxonomic, and genotypic data obtained in this study show that MAFF 302110^T^/MAFF 302107 represent a novel species of the genus *Pectobacterium*, for which we propose the name *Pectobacterium araliae* sp. nov., designating MAFF 302110^T^ (=ICMP 25161^T^) as the type strain.

## Introduction

The genus *Pectobacterium*, belonging to the family *Pectobacteriaceae* of the order *Enterobacterales*, encompasses a diverse group of Gram-negative, rod-shaped, pectinolytic bacteria responsible for soft rot diseases in a wide array of plant hosts [1,6]. These bacteria are characterized by their ability to secrete a vast repertoire of pectinolytic and cellulolytic enzymes that break down plant cell walls, leading to characteristic soft mushy lesions associated with soft rot diseases. They have a global distribution and exert a significant impact on agriculture, causing substantial economic losses because of reduced yields and crop production [7,8]. Whole-genome sequencing technologies have driven recent revisions to the taxonomy of the order *Enterobacterales*, including the genus *Pectobacterium*, leading to the reclassifications and recognition of new species [2,6,9]. At the time of writing this paper, 20 species with validly published and correct names existed in the genus *Pectobacterium* according to the List of Prokaryotic names with Standing in Nomenclature (https://lpsn.dsmz.de) [10].

The Japanese angelica tree, or Japanese aralia (*Aralia elata*), is a deciduous shrub of the family *Araliaceae* that is distributed in Japan and eastern Asia. In Japan, it has long been customary to eat its new shoots and the trees have been cultivated extensively, especially since the 1980s. In 1987, soft rot symptoms were observed in the new shoots and stems of Japanese angelica trees cultivated in Yamanashi Prefecture, Japan (Fig. S1A, available in the online version of this article). The affected shoots turned blackish brown, the stems turned brown and decayed, and the decaying parts eventually died. In some cases, soft rot symptoms also occurred in the roots of intensely affected trees [11].

Ono *et al*. [11] isolated a causative bacterium from the diseased tissues, confirmed its pathogenicity in Japanese angelica trees (Fig. S1B), and investigated its phenotypic characteristics. Based on these results, the causative bacterium was identified as *Erwinia carotovora* subsp. *carotovora* and the disease was named ‘bacterial soft rot of Japanese angelica tree’ [12]. However, following subsequent major changes in the classification of the order *Enterobacterales* [1,6, 9], which also includes the genus *Erwinia*, a preliminary re-examination of the taxonomic affiliation of the causative bacterium deposited in the Genebank Project (www.gene.affrc.go.jp/databases-micro_search_en.php) of the National Agriculture and Food Research Organization (NARO), Japan, was performed. These results suggested that the causative bacterium may not belong to the genus *Erwinia*, but to a new species of the genus *Pectobacterium*.

The objective of this study was to clarify the taxonomic affiliation of MAFF 302110^T^ (=ICMP 25161^T^) and MAFF 302107 (=ICMP 25162), both of which are the pathogen of soft rot disease of Japanese angelica tree, identified as *E. carotovora* and deposited in the NARO Genebank, Japan, by Ono *et al*. [11]. To this end, an extensive comparative analysis of these strains in conjunction with related species was conducted using a polyphasic approach. Our findings indicate that they are members of the genus *Pectobacterium* and represent a novel species that we propose to name *Pectobacterium araliae* sp. nov.

## Genome features

For the whole genome sequencing, MAFF 302110^T^ and MAFF 302107 were cultured in Luria–Bertani broth for 18 h at 30 °C while shaking and their total genomic DNA was extracted using a NucleoSpin Tissue Kit (Takara Bio) following the manufacturer’s protocol. The complete genome sequence of MAFF 302110^T^ was obtained using a PacBio Revio (Pacific Biosciences) using libraries prepared with a SMRTbell gDNA Sample Amplification Kit and SMRTbell Express Template Prep Kit 2.0 (Pacific Biosciences), from Seibutsu Giken Inc. (Kanagawa, Japan). Sequencing reads were assembled using Canu version 1.9 [13] and Flye version 2.9.2 [14]. To obtain the draft genome sequence of MAFF 302107, library construction and sequencing, using the HiSeq X Ten platform (Illumina), were performed by Eurofins Genomics Inc. (Tokyo, Japan). Adapter-trimmed raw reads were assembled using SPAdes version 3.13.0 [15]. The assemblies obtained (Table S1) were annotated using the DDBJ Fast Annotation and Submission Tool (DFAST; https://dfast.ddbj.nig.ac.jp), which is a prokaryotic genome annotation pipeline [16]. Briefly, protein-coding sequences were predicted using MetaGeneAnnotator [17]. Genes encoding tRNA and rRNA were identified using Aragorn version 1.2.38 [18] and Barrnap version 0.8 (https://github.com/tseemann/barrnap), respectively.

The genome of MAFF 302110^T^ included one circular chromosome but no indigenous plasmids. The chromosome was 4 663 678 bp in size, with a G+C content of 51.1 mol% and contained 4124 protein-coding sequences, 22 rRNA genes, and 76 tRNA genes. The G+C content was within the range of 50.5–56.1 mol% reported for the *Pectobacterium* species [19]. The genome sequencing data obtained for MAFF 302110^T^ and MAFF 302107 were deposited in DDBJ/ENA/GenBank under accession numbers AP028908 and BRCR00000000, respectively (Table S1).

To taxonomically evaluate the relationship between MAFF 302110^T^ and MAFF 302107, digital DNA–DNA hybridization (dDDH) analysis was performed using their genome sequences. For this purpose, formula 2 of the Genome-to-Genome Distance Calculator 3.0 (GGDC 3.0; https://ggdc.dsmz.de/ggdc.php) [20,21] was used. As a result, a high value of 98.8 % was obtained between the two strains, which was well above the threshold (70 %) for the delineation of prokaryotic species [22], indicating that they are included in the same species framework.

To preliminarily assess the taxonomic affiliation of MAFF 302110^T^ by genome analysis, the Type Strain Genome Server (TYGS; https://tygs.dsmz.de) [23] and Taxonomy Check implemented in DFAST [16] were used. The MAFF 302110^T^ genome sequence was uploaded to TYGS and the dDDH indices were calculated against the type strain genomes to identify the closest type strain. The results showed that although the dDDH value calculated for *Pectobacterium polonicum* DPMP315^T^ was the highest (47.1 %; Table S2), it was below the cut-off value for the prokaryotic species delineation [22]. In a taxonomy check using the fast average nucleotide identity (FastANI) algorithm [24] (Table S3), the closest strain was *P. polonicum* DPMP315^T^, but its value (92.224 % ANI) was also lower than the threshold (95–96 % ANI) for the prokaryotic species delineation [22]. These results suggest that MAFF 302110^T^ and MAFF 302107 may represent a novel species of the genus *Pectobacterium*.

## 16s rRNA gene analyses

To comprehensively identify the bacterial species closely related to MAFF 302110^T^/MAFF 302107, a homology search based on 16S rRNA gene sequences was performed as described previously [25,26]. Briefly, their partial sequences were determined by direct sequencing of the PCR products, and a homology search was conducted in the EzBioCloud database (www.ezbiocloud.net/identify) [27] using the sequence of MAFF 302110^T^ as a query. A total of 43 known species showing high similarity to MAFF 302110^T^ were identified, and their 16S rRNA gene sequences were collected from the EzBioCloud database. Next, the 16S rRNA gene sequences of the species that exhibited high dDDH/ANI values with MAFF 302110^T^ in the aforementioned genome analyses performed using TYGS and DFAST (Tables S2 and S3) were retrieved from GenBank. Using a pairwise nucleotide sequence alignment tool (www.ezbiocloud.net/tools/pairAlign) [27] in EzBioCloud, the similarity values against the 16S rRNA gene sequence of MAFF 302110^T^ were calculated. Finally, by combining the data derived from EzBioCloud, TYGS, and DFAST and eliminating duplicates, 45 known species with validly published and correct names were selected as closely related to MAFF 302110^T^/MAFF 302107 (Table S4). Among these, *Pectobacterium quasiaquaticum* A477-S1-J17^T^ showed the highest sequence similarity (99.36 %) to MAFF 302110^T^.

To determine the phylogenetic position of MAFF 302110^T^/MAFF 302107, phylogenetic analyses based on 16S rRNA gene sequences were carried out as described in our previous studies [25,26], targeting MAFF 302110^T^/MAFF 302107, the 45 species (selected as closely related species of MAFF 302110^T^/MAFF 302107; Table S4), and *Budvicia aquatica* DSM 5075^T^ (selected as an outgroup based on the analysis result of Adeolu *et al*. [9]). The 16S rRNA gene sequences were analysed using mega 11 version 11.0.13 [28] with the neighbour-joining, maximum-likelihood, and maximum-parsimony methods. The reliability of the tree was tested using the standard bootstrap method with 1000 replications. In the resulting phylogenetic trees, MAFF 302110^T^ and MAFF 302107 clustered as a monophyletic clade with a bootstrap value of 99 % (Fig. S2). However, the phylogenetic position of this clade did not match any of the known species used in the analyses.

## *Gyrb* gene analyses

The phylogenetic position of MAFF 302110^T^/MAFF 302107 was evaluated using the *gyrB* gene sequences of the same members as those used in the 16S rRNA gene sequence analyses (Fig. S2). The partial sequences of MAFF 302110^T^/MAFF 302107 were determined by direct PCR sequencing using the primers described by Brady *et al*. [29], whereas those of the 45 related species (Table S4) and *Budvicia aquatica* DSM 5075^T^ (outgroup) were extracted from the respective whole genome sequences. Phylogenetic analyses were performed using these sequences, as described previously [30]. The resulting phylogenetic trees revealed that MAFF 302110^T^ and MAFF 302107 were tightly clustered (Fig. S3), which was similar to the results of the 16S rRNA gene sequence analyses (Fig. S2). Furthermore, these two strains were found to cluster together with the known species of the genus *Pectobacterium* as a monophyletic clade with a standard bootstrap value of 100 %. However, the phylogenetic position of MAFF 302110^T^/MAFF 302107 was not consistent with that of any member of the genus.

## Comparative genomics and phylogenomics

A comprehensive analysis of whole-genome similarity was conducted by calculating ANI and dDDH values between MAFF 302110^T^ and closely related species. To calculate the ANI values, two different methods were employed: ANI algorithm using blast (ANIb; http://enve-omics.ce.gatech.edu/g-matrix/index) [31] and orthologous ANI algorithm using usearch (OrthoANIu; www.ezbiocloud.net/tools/ani) [32]. The dDDH values were determined using formula 2 of GGDC 3.0 (https://ggdc.dsmz.de/ggdc.php) [20,21]. The genome sequence of MAFF 302110^T^ was used as a query and the 45 closely related species (Table S4) were selected for comparison.

Table 1 presents the ANI and dDDH values obtained for these species arranged in descending order based on their ANIb values calculated against the MAFF 302110^T^ genome sequence. The ANIb values between MAFF 302110^T^ and its closely related species ranged from 77.91 % (in the case of *Huaxiibacter chinensis* 155047^T^) to 91.74 % (in the case of *P. polonicum* DPMP315^T^), while the OrthoANIu values varied between 72.98 % (for *H. chinensis* 155047^T^) and 92.10 % (for *P. polonicum* DPMP315^T^). Notably, both ANI values were below the established cut-off for the prokaryotic species delineation [22]. Additionally, the dDDH values ranged from 20.0 % (for *Enterobacter soli* LMG 25861^T^) to 47.1 % (for *P. polonicum* DPMP315^T^), which fell below the recognized threshold for prokaryotic species delineation [22].

**Table 1.** Genomic relationship between strain MAFF 302110^T^ and type strains of closely related species The ANIb, OrthoANIu, and dDDH values were calculated using the genome-based distance matrix calculator [31], the ANI calculator [32], and the Genome-to-Genome Distance Calculator 3.0 (formula 2) [20,21], respectively. The data shown here have been presented in descending order of their ANIb values calculated against the MAFF 302110^T^ genome sequence.

Species	Strain	Accession no.*	ANIb (%)	OrthoANIu (%)	dDDH (%)
***Pectobacterium araliae***	MAFF 302110^T^	AP028908.1	100.00	100.00	100.0
***Pectobacterium araliae***	MAFF 302107	BRCR00000000.1	99.91	99.85	98.8
*Pectobacterium polonicum*	DPMP315^T^	RJTN00000000.1	91.74	92.10	47.1
*Pectobacterium punjabense*	SS95^T^	NZ_CP038498.1	90.65	91.12	43.8
*Pectobacterium carotovorum*	NCPPB 312^T^	JQHJ00000000.1	89.53	90.06	40.1
*Pectobacterium versatile*	CFBP 6051^T^	RMBU00000000.1	89.49	90.12	39.9
*Pectobacterium wasabiae*	CFBP 3304^T^	NZ_CP015750.1	89.43	89.75	40.0
*Pectobacterium parmentieri*	RNS 08-42-1A^T^	NZ_CP015749.1	89.42	89.75	39.9
*Pectobacterium odoriferum*	NCPPB 3839^T^	JQOG00000000.1	89.38	89.97	39.8
*Pectobacterium aquaticum*	A212-S19-A16^T^	NZ_CP086253.1	88.99	89.49	38.6
*Pectobacterium actinidiae*	KKH3^T^	JRMH00000000.1	88.84	89.39	38.3
*Pectobacterium polare*	NIBIO1006^T^	NZ_CP017481.1	88.83	89.42	38.4
*Pectobacterium parvum*	s0421^T^	OANP00000000.3	88.81	89.32	38.2
*Pectobacterium quasiaquaticum*	A477-S1-J17^T^	NZ_CP065177.1	88.60	89.19	37.7
*Pectobacterium brasiliense*	LMG 21371^T^	JQOE00000000.1	88.51	89.16	37.4
*Pectobacterium atrosepticum*	NCPPB 549^T^	JQHK00000000.1	88.35	89.00	37.1
*Pectobacterium peruviense*	IFB5232^T^	LXFV00000000.1	88.31	88.95	37.1
*Pectobacterium betavasculorum*	NCPPB 2795^T^	JQHM00000000.1	88.01	88.64	36.2
*Pectobacterium aroidearum*	L6	NZ_CP065044.1	87.71	88.48	35.8
*Pectobacterium fontis*	M022^T^	JSXC00000000.1	86.78	87.58	33.5
*Leclercia pneumoniae*	4-9-1-25^T^	NZ_CP071383.1	79.44	73.17	20.9
*Klebsiella pneumoniae* subsp*. ozaenae*	NCTC 5050^T^	UGLZ00000000.1	79.41	73.26	22.5
*Symbiopectobacterium purcellii*	SyEd1^T^	NZ_CP081864.1	79.36	75.28	21.0
*Serratia entomophila*	A1^T^	NZ_CP082787.1	79.22	74.97	21.3
*Lelliottia jeotgali*	PFL01^T^	CP018628.1	79.18	73.42	21.4
*Yokenella regensburgei*	NCTC 11966^T^	UHJH00000000.1	79.13	73.12	21.5
*Scandinavium goeteborgense*	CCUG 66741^T^	NZ_CP054058.1	79.09	73.36	21.6
*Klebsiella aerogenes*	KCTC 2190^T^	NC_015663.1	79.06	73.23	21.8
*Klebsiella africana*	200023^T^	NZ_CP084874.1	78.99	73.28	21.9
*Serratia ficaria*	NCTC 12148^T^	NZ_LT906479.1	78.95	74.69	21.0
*Dickeya lacustris*	S29^T^	QNUT00000000.1	78.88	74.67	20.7
*Enterobacter bugandensis*	EB-247^T^	NZ_LT992502.1	78.88	73.55	21.1
*Dickeya chrysanthemi*	NCPPB 402^T^	AOOA00000000.1	78.76	75.13	20.5
*Klebsiella pasteurii*	SB6412^T^	CABGHC000000000.1	78.74	73.08	21.1
*Serratia ureilytica*	CCUG 50595^T^	JABXOF000000000.1	78.58	74.84	21.0
*Leclercia tamurae*	H6S3^T^	JAMHKS000000000.1	78.56	73.09	20.8
*Klebsiella pneumoniae* subsp*. rhinoscleromatis*	ATCC 13884^T^	ACZD00000000.1	78.51	73.20	21.4
*Enterobacter wuhouensis*	WCHEW120002^T^	SJOO00000000.1	78.49	73.43	20.6
*Klebsiella quasipneumoniae* subsp*. similipneumoniae*	07 A044^T^	CBZR000000000.1	78.47	73.13	21.1
*Raoultella planticola*	ATCC 33531^T^	JMPP00000000.1	78.44	73.29	21.5
*Enterobacter cancerogenus*	ATCC 33241^T^	FYBA00000000.1	78.39	73.31	20.7
*Klebsiella quasipneumoniae* subsp*. quasipneumoniae*	01 A030^T^	CCDF00000000.1	78.32	73.12	20.9
*Enterobacter soli*	LMG 25861^T^	FYBB00000000.1	78.24	73.08	20.0
*Silvania hatchlandensis*	H19S6^T^	JAMGZK000000000.1	78.24	73.17	20.3
*Klebsiella variicola* subsp*. tropica*	SB5531^T^	CAAHGN000000000.1	78.12	73.23	21.3
*Raoultella ornithinolytica*	NBRC 105727^T^	BCYR00000000.1	78.12	73.14	21.3
*Huaxiibacter chinensis*	155047^T^	JAJVHF000000000.1	77.91	72.98	20.3

**Fig. 1.**
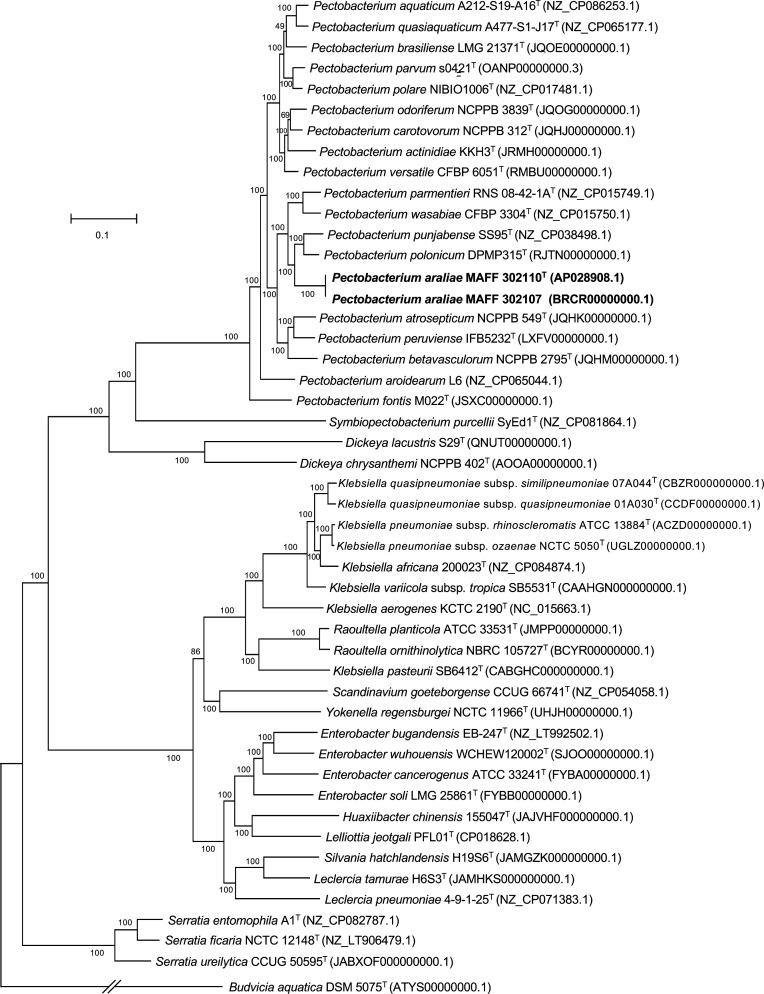
Phylogenomic tree reconstructed based on the concatenated alignment of 807 core genes (total alignment length of 639 079 bp), showing the relationships between *Pectobacterium araliae* sp. nov. strains (boldface type) and the closely related species listed in Table 1. The concatenated alignment was generated using Roary [34] and Gblocks [36]. The maximum-likelihood tree was inferred using RAxML-NG [37] with a general time-reversible substitution model and a gamma model of rate heterogeneity. *Budvicia aquatica* DSM 5075^T^ served as an outgroup. GenBank accession numbers are shown in parentheses. The numbers at the nodes indicate standard bootstrap values from 100 replications.

*The whole genome sequences used here are the same as those used in the phylogenomic analysis (Fig. 1).

To further elucidate the phylogenetic placement of MAFF 302110^T^/MAFF 302107, a comprehensive phylogenomic analysis was performed based on the concatenated alignment of core genes that constitute the core genome of MAFF 302110^T^/MAFF 302107, the 45 closely related species (Table 1), and *Budvicia aquatica* DSM 5075^T^ (an outgroup). Genomic annotations were generated *de novo* using Prokka [33] and the annotated genes were compared across all input genomes using the Roary pan-genome analysis pipeline [34], applying a 70 % amino acid identity threshold. This analysis identified 807 core genes that were present in all the genomes under investigation. Subsequently, the nucleotide sequences of these core genes were concatenated, and multiple alignments were conducted using mafft [35] implemented through Roary. To enhance the quality of the concatenated alignment, poorly aligned positions and divergent regions were removed using Gblocks [36] using default parameters. From the resulting alignment, which had a total length of 639 079 bp, a maximum-likelihood tree was reconstructed using RAxML-NG [37], with a general time-reversible substitution model and a gamma model of rate heterogeneity. To evaluate the reliability of the tree, the standard bootstrap method with 100 replicates was used. The resulting phylogenomic tree positioned MAFF 302110^T^/MAFF 302107 within the clade of the genus *Pectobacterium* (Fig. 1). However, it is important to note that the phylogenetic placement of MAFF 302110^T^/MAFF 302107 did not correspond to that of any other member of the genus.

## Physiology and chemotaxonomy

The phenotypic characteristics of MAFF 302110^T^/MAFF 302107 were compared with those of four *Pectobacterium* species (*P. polonicum*, *P. parmentieri*, *P. punjabense*, and *P. wasabiae*) that were closely related to MAFF 302110^T^/MAFF 302107 in the phylogenomic analysis (Fig. 1). The data for the latter four species were retrieved from Waleron *et al*. [4,5] and Khayi *et al*. [2] for comparison. MAFF 302110^T^/MAFF 302107 were cultured routinely at 28 °C on standard methods agar (plate count agar) plates (Nissui) and tested for the phenotypic characteristics, as described below. Cell size, morphology, and flagellar insertion were determined using transmission electron microscopy as described previously [25]. Colony morphology and growth at 37 °C were evaluated using TY (tryptone–yeast extract) agar plates [5 g l^−1^ tryptone, 3 g l^−1^ yeast extract, and 1.5 % (w/v) agar] [2]. Gram reaction (Ryu non-staining KOH method), motility, oxidase activity, and potato soft rot were examined as described by Schaad *et al*. [38]. Catalase activity was determined as described by Lelliott *et al*. [39]. Carbon source utilization and chemical sensitivity were evaluated using the Biolog GEN III MicroPlate system (Biolog) according to the manufacturer’s protocol with the following modifications [25,40]. The results of the Biolog assays were scored after 3 days of incubation at 28 °C. The Biolog assays were repeated thrice, and only the stable results are shown in Table 2 and the species description below.

**Table 2.** Characteristics that differentiate *Pectobacterium araliae* sp. nov. strains from the closely related *Pectobacterium* species Strains: 1-1, *Pectobacterium araliae* MAFF 302110^T^ (this study); 1-2, *Pectobacterium araliae* MAFF 302107 (this study); 2, *Pectobacterium polonicum* DPMP315^T^ (data from [5]); 3, *Pectobacterium parmentieri* SCC3193 [2, 4, 5]; 4, *Pectobacterium punjabense* IFB5596 [5]; 5, *Pectobacterium wasabiae* CFBP 3304^T^ [4, 5]. Characteristics have been scored as follows: +, positive reaction; –, negative reaction; w, weakly positive reaction; na, not available.

Characteristic	1-1	1-2	2	3	4	5
Catalase	+	w	na	+	na	+
Potato soft rot	+	+	+	+	+	w
Growth at 37 °C	w	w	+	+	+	+
Growth at pH 5	–	–	–	+	+	w
Utilization of (Biolog GEN III):*						
Stachyose	–	–	+	+	–	–
Raffinose	–	–	+	+	na	+
Melibiose	–	–	+	+	na	–
*N*-Acetyl-d-galactosamine	–	–	–	+	–	–
Inosine	–	–	–	–	w	+
d-Glucose-6-phosphate	–	–	+	+	na	+
d-Aspartic acid	+	+	+	+	na	w
d-Serine	–	–	–	w	na	–
Gelatin	–	–	–	–	–	+
l-Glutamic acid	+	+	+	+	–	+
d-Glucuronic acid	+	+	–	–	–	+
Glucuronamide	+	+	–	–	–	w
l-Lactic acid	+	+	+	–	–	+
Citric acid	+	+	+	+	–	–
α-Hydroxybutyric acid	–	–	–	–	na	+
Formic acid	+	+	w	–	+	+
Isolation source	Japanese angelica tree (*Aralia elata*)	Japanese angelica tree (*Aralia elata*)	Groundwater	Potato (*Solanum tuberosum*)	Potato (*Solanum tuberosum*)	Wasabi (*Eutrema japonicum*)
Genome size (Mb)†	4.66	4.65	4.84	5.16	4.90	5.04
DNA G+C content (mol%)†	51.1	50.9	51.3	50.4	51.0	50.6

*The results were scored after incubation for 3 days at 28°C.

†These results were calculated from the respective genome DNA sequences.

MAFF 302110^T^ was rod-shaped with peritrichous flagella (Fig. S4). The cell size (mean±standard deviation) was 2.1±0.5×0.9±0.08 µm (*n*=25). On TY agar plates, MAFF 302110^T^/MAFF 302107 grew well at 28 °C, but showed weak growth at 37 °C. The strains formed visible colonies after incubation for 24 h at 28 °C on TY agar plates. The colonies were approximately 1–2 mm in diameter after 48 h and 6 mm in diameter (maximum) after approximately 6 days. Additionally, the characteristics of the colonies were as follows: pale yellow to greyish-white in colour, opaque, round with entire margins, raised, smooth, and glistening (Fig. S5). No diffusible pigments were observed when the cells were cultured on TY agar plates. MAFF 302110^T^/MAFF 302107 were Gram-reaction-negative, facultatively anaerobic, and exhibited positive responses for motility, potato soft rot, and growth at pH 6. However, these strains exhibited negative responses for oxidase activity and growth at pH 5. Different responses were observed between MAFF 302110^T^ and MAFF 302107 for catalase activity (Table 2). In the Biolog assays, the strains utilized trehalose, cellobiose, gentiobiose, sucrose, lactose, methyl β-d-glucoside, d-salicin, *N*-acetyl-d-glucosamine, α-d-glucose, d-mannose, d-fructose, d-galactose, l-rhamnose, d-mannitol, *myo*-inositol, glycerol, d-aspartic acid, l-aspartic acid, l-glutamic acid, pectin, d-galacturonic acid, l-galactonic acid lactone, d-gluconic acid, d-glucuronic acid, glucuronamide, mucic acid, d-saccharic acid, l-lactic acid, citric acid, l-malic acid, bromosuccinic acid, acetic acid, and formic acid, but failed to utilize maltose, stachyose, raffinose, melibiose, *N*-acetyl-d-galactosamine, *N*-acetyl neuraminic acid, 3-methyl glucose, d-fucose, l-fucose, inosine, d-sorbitol, d-glucose-6-phosphate, d-serine, gelatin, glycyl-l-proline, l-alanine, l-arginine, l-histidine, l-pyroglutamic acid, quinic acid, *p*‐hydroxyphenylacetic acid, d-lactic acid methyl ester, γ-aminobutyric acid, α-hydroxybutyric acid, dl-β-hydroxybutyric acid, and α-ketobutyric acid. The Biolog assays indicated that the strains were resistant to fusidic acid, troleandomycin, rifamycin SV, niaproof 4, vancomycin, tetrazolium violet, tetrazolium blue, and potassium tellurite, while susceptible to d-serine, minocycline, and sodium bromate. The following phenotypic characteristics of MAFF 302110^T^/MAFF 302107 could distinguish them from the closest relatives (Table 2): positive for potato soft rot and utilization of d-aspartic acid, l-glutamic acid, d-glucuronic acid, glucuronamide, l-lactic acid, citric acid, and formic acid; weakly positive for growth at 37 °C; and negative for growth at pH 5 and utilization of stachyose, raffinose, melibiose, *N*-acetyl-d-galactosamine, inosine, d-glucose-6-phosphate, d-serine, gelatin, and α-hydroxybutyric acid.

To conduct cellular fatty acid analysis, MAFF 302110^T^ was cultured in minimal medium M9 [41] for 48 h at 28 °C while shaking. Subsequently, cellular fatty acids were prepared and analysed as described in our previous studies [25,26], utilizing the Sherlock Microbial Identification System version 6.0 (midi) and TSBA6 method. The major fatty acids, those constituting more than 5 % of the total fatty acids in MAFF 302110^T^ (Table 3), included summed feature 3 (31.6 %), C_16 : 0_ (26.4 %), summed feature 8 (15.9 %), summed feature 2 (14.8 %), and C_12 : 0_ (9.6 %). The composition ratios of these major fatty acids were similar between MAFF 302110^T^ and the closely related *Pectobacterium* species. Nevertheless, some variations were observed in the minor fatty acid species (Table 3). Notably, C_15 : 0_ was detected exclusively in *P. wasabiae*, while C_17 : 0_ was absent in MAFF 302110^T^.

**Table 3.** Cellular fatty acid composition (as percentages of the total) of strain MAFF 302110^T^ and the closely related *Pectobacterium* species Strains: 1, MAFF 302110^T^; 2, *Pectobacterium polonicum* DPMP315^T^; 3, *Pectobacterium wasabiae* CFBP 3304^T^. Data for strain MAFF 302110^T^ were obtained in this study. The other data were from Waleron *et al.* [5], under the same culture and analytical conditions used in this study. –, Not detected. Fatty acids representing <1 % in all strains are not shown. The major fatty acids of each species (>5 % of the total fatty acids) are highlighted in bold.

Fatty acid	1	2	3
C_12 : 0_	**9.6**	**6.0**	**5.7**
C_14 : 0_	1.8	1.2	1.4
C_15 : 0_	–	–	3.0
C_16 : 0_	**26.4**	**27.0**	**25.9**
C_17 : 0_	–	2.5	1.7
Summed feature 2*	**14.8**	**8.8**	**7.5**
Summed feature 3†	**31.6**	**29.9**	**33.2**
Summed feature 8‡	**15.9**	**21.3**	**16.5**

*Summed feature 2 comprises any combination of C_12:0_ aldehyde, an unknown fatty acid with an equivalent chain length of 10.928, C_16:1_ iso I, and C_14:0_ 3OH.

†Summed feature 3: C_16:1_ ω7*c* and/or C_16:1_ ω6*c*.

‡Summed feature 8: C_18:1_ ω7*c* and/or C_18:1_ ω6*c.*

## Functional genomics

Functional annotation and metabolic reconstruction of the genome of MAFF 302110^T^ were performed using BlastKOALA and kegg Mapper from the Kyoto Encyclopedia of Genes and Genomes (kegg; www.kegg.jp/kegg/) [42]. The kegg annotation predicted that the MAFF 302110^T^ genome encoded 87 complete pathway modules that were organized into the following functional subcategories in the metabolism category: carbohydrate metabolism, energy metabolism, lipid metabolism, nucleotide metabolism, amino acid metabolism, glycan metabolism, metabolism of cofactors and vitamins, biosynthesis of terpenoids and polyketides, and biosynthesis of other secondary metabolites. The carbohydrate metabolism genes (338 genes) were the most abundant in the genome (Table S5). Genes encoding flagellar biosynthesis and assembly proteins, including *flgABCDEFGHIJKLMNYZ*, *flhABCDE*, *fliACDEFGHIJKLMNOPQRST*, and *motAB*, were predicted to be present in the genome, which is in accordance with the observation that MAFF 302110^T^ was motile with peritrichous flagella (Fig. S4). Additionally, numerous genes potentially involved in membrane transport (including ABC transporters, phosphotransferase systems, and bacterial secretion systems; 251 genes), signal transduction (including two-component systems; 131), cellular community (including quorum sensing and biofilm formation; 126), and bacterial chemotaxis (19) were detected (Table S5), suggesting that MAFF 302110^T^ may be sensitive to various environmental stimuli.

Several factors, some of which are given below, may be involved in the pathogenicity of *Pectobacterium* species to plants [43,46]: two-component signal transduction systems, quorum-sensing, secretion systems, carbohydrate-active enzymes (CAZymes) including plant cell wall-degrading enzymes, toxins, and siderophores. Of these, CAZymes and secretion systems are considered to be key virulence factors [47,48]. Therefore, MAFF 302110^T^ and the type strains of the closely related *Pectobacterium* species were compared based on these two factors. Based on their catalytic activity and amino acid sequence similarity, CAZymes are divided into several classes, including auxiliary activities (AAs), carbohydrate-binding modules (CBMs), carbohydrate esterases (CEs), glycoside hydrolases (GHs), glycosyl transferases (GTs), and polysaccharide lyases (PLs). To assess the number of domains encoding putative CAZymes for each of the above classes, genome mining was performed using the dbCAN3 meta-server (https://bcb.unl.edu/dbCAN2/index.php) [49]. If an annotation was supported by at least two of the following tools/databases in dbCAN3: diamond (CAZy), hmmer (dbCAN), or hmmer (dbCAN-sub), the hit was assigned to the corresponding CAZyme class. For the secretion systems, the total number of genes assigned to the core components of each system by kegg annotation was calculated for each strain and compared among the strains.

CAZyme analysis using dbCAN3 predicted 111 domains encoding six major classes of CAZymes in the MAFF 302110^T^ genome (Table S6). Among the putative CAZymes, GHs were the most abundant (47 domains) in the genome, followed by GTs (30), PLs (13), CBMs (11), CEs (8), and AAs (2). A number of putative CAZyme domains were also identified in the genomes of the closely related species, whose CAZyme profiles were quite similar to those of MAFF 302110^T^ (Table S6). The large and diverse set of CAZyme domains identified in the genomes of the strains investigated suggests that these enzymes may play a crucial role in their pathogenic mechanisms and/or ecology.

Among the secretion systems, the number of genes assigned to the core components of the Type II, Sec, and Tat systems differed slightly among the strains (Table S7). The high conservation of these systems in the strains suggests that they might play a critical role in the pathogenicity of the strains, which is consistent with the previous reports that a variety of CAZymes degrading plant cell walls are secreted via the Type II secretion system [43,44, 47, 48]. In contrast, for Type III (excluding the flagellar secretion system), IV, and VI secretion systems, the number of genes assigned varied widely among the strains (Table S7). Type IV secretion system genes were detected in MAFF 302110^T^, *P. parmentieri*, and *P. wasabiae*. However, no Type III genes were detected. *P. polonicum* and *P. punjabense* were the opposite of these three strains, with Type III secretion system genes detected, but no Type IV genes. For the Type VI secretion system, no relevant core genes were found at all in *P. punjabense*. Among the other four strains, the numbers of Type VI genes differed significantly. The diversity observed among the strains with regard to the Type III, IV, and VI secretion systems (Table S7) may have a significant impact on the pathogenicity, virulence, host range, and/or ecology of these strains. However, to clarify this point, not only the core components of the secretion systems, but also the effectors secreted via these systems need to be comprehensively analysed and compared among the strains.

## Taxonomic conclusion

Phylogenetic analyses of 16S rRNA and *gyrB* gene sequences (Figs S2 and S3) and examination of the cellular fatty acid composition (Table 3) and G+C content (Table 2), as well as the preliminary genome analyses carried out at TYGS and DFAST (Tables S2 and S3), consistently suggested the affiliation of MAFF 302110^T^/MAFF 302107 with the genus *Pectobacterium*. Phylogenomic analysis using whole-genome sequences revealed that the phylogenetic placement of the strains did not align with any known species within the genus (Fig. 1). The results of the ANIb, OrthoANIu, and dDDH analyses (Table 1) confirmed that MAFF 302110^T^/MAFF 302107 represent a novel species of the genus *Pectobacterium*, for which we propose the name *Pectobacterium araliae* sp. nov., designating MAFF 302110^T^ (=ICMP 25161^T^) as the type strain. Distinguishing features of *Pectobacterium araliae* sp. nov. from its closest relatives were evident in its phenotypic characteristics (Table 2), cellular fatty acid composition (Table 3), and repertoire of secretion systems (Table S7).

## Description of *Pectobacterium araliae* sp. nov.

*Pectobacterium araliae* (a.ra'li.ae. N.L. gen. n. *araliae*, of the Japanese angelica tree *Aralia elata*).

Members of this species are Gram-reaction-negative, facultatively anaerobic, motile with peritrichous flagella, rod-shaped, and non-spore-forming. The cell size (mean±standard deviation) is 2.1±0.5×0.9±0.08 µm (*n*=25). On TY agar plates, grows well at 28 °C, forming visible colonies within 24 h, but the growth is weak at 37 °C. Colonies are pale yellow to greyish-white in colour, opaque, round with entire margins, raised, smooth, and glistening, with a diameter of approximately 1–2 mm, after culturing on TY agar plates for 48 h at 28 °C. No diffusible pigments are observed when cultured on the plates. The strains of the species are positive for potato soft rot and growth at pH 6, but negative for oxidase activity and growth at pH 5. Different catalase activity responses are observed between the strains.

Plate test analysis performed using the Biolog GEN III MicroPlate system (3 days incubation at 28 °C) indicates that the species utilizes trehalose, cellobiose, gentiobiose, sucrose, lactose, methyl β-d-glucoside, d-salicin, *N*-acetyl-d-glucosamine, α-d-glucose, d-mannose, d-fructose, d-galactose, l-rhamnose, d-mannitol, *myo*-inositol, glycerol, d-aspartic acid, l-aspartic acid, l-glutamic acid, pectin, d-galacturonic acid, l-galactonic acid lactone, d-gluconic acid, d-glucuronic acid, glucuronamide, mucic acid, d-saccharic acid, l-lactic acid, citric acid, l-malic acid, bromosuccinic acid, acetic acid, and formic acid; but fails to utilize maltose, stachyose, raffinose, melibiose, *N*-acetyl-d-galactosamine, *N*-acetyl neuraminic acid, 3-methyl glucose, d-fucose, l-fucose, inosine, d-sorbitol, d-glucose-6-phosphate, d-serine, gelatin, glycyl-l-proline, l-alanine, l-arginine, l-histidine, l-pyroglutamic acid, quinic acid, *p*‐hydroxyphenylacetic acid, d-lactic acid methyl ester, γ-aminobutyric acid, α-hydroxybutyric acid, dl-β-hydroxybutyric acid, and α-ketobutyric acid. In the Biolog GEN III assays, the strains are resistant to fusidic acid, troleandomycin, rifamycin SV, niaproof 4, vancomycin, tetrazolium violet, tetrazolium blue, and potassium tellurite; while susceptible to d-serine, minocycline, and sodium bromate.

The major cellular fatty acids (>5  % of the total fatty acids) in MAFF 302110^T^ are summed feature 3 (C_16 : 1_ *ω*7*c* and/or C_16 : 1_ *ω*6*c*), C_16 : 0_, summed feature 8 (C_18 : 1_ *ω*7*c* and/or C_18 : 1_ *ω*6*c*), summed feature 2 (comprising any combination of C_12 : 0_ aldehyde, an unknown fatty acid with an equivalent chain length of 10.928, C_16 : 1_ iso I, and C_14 : 0_ 3OH), and C_12 : 0_. The genomic DNA G+C content of MAFF 302110^T^ is 51.1 mol % and its genomic size is approximately 4.66 Mbp.

The type strain, MAFF 302110^T^ (=ICMP 25161^T^), was isolated from a lesion formed on a Japanese angelica tree (*Aralia elata*) affected by bacterial soft rot, which was sampled in Yamanashi Prefecture, Japan, in 1987, and is pathogenic to a Japanese angelica tree. MAFF 302107 (=ICMP 25162) is an additional strain of this species.

The genome sequencing data for MAFF 302110^T^ and MAFF 302107 have been deposited in DDBJ/ENA/GenBank under accession numbers AP028908 and BRCR00000000, respectively. The 16S rRNA gene sequences of MAFF 302110^T^ and MAFF 302107 are under accession numbers LC773403 and LC773404, respectively.

## supplementary material

10.1099/ijsem.0.006326 Uncited Supplementary Material 1.

## Abbreviations

ANIb: average nucleotide identity algorithm using blast
CAZyme: carbohydrate-active enzyme
dDDH: digital DNA–DNA hybridization
DFAST: DDBJ Fast Annotation and Submission Tool
GGDC: Genome-to-Genome Distance Calculator
KEGG: Kyoto Encyclopedia of Genes and Genomes
OrthoANIu: orthologous ANI algorithm using USEARCH
TY: tryptone–yeast extract
TYGS: Type Strain Genome Server
