# Supplementary material for: Pectobacterium araliae sp. nov., a pathogen causing bacterial soft rot of Japanese angelica tree in Japan
Source: Int J Syst Evol Microbiol. 2024 Apr 16;74(4):006326. doi: 10.1099/ijsem.0.006326 (PMC11092084; doi:10.1099/ijsem.0.006326)
Supplement: Uncited Supplementary Material 1. [file ijsem-74-06326-s001.pdf]

IJSEM supplementary materials

Pectobacterium araliae sp. nov., a pathogen causing bacterial soft rot of Japanese angelica tree in Japan

Hiroyuki Sawada[1*], Nobutaka Someya[2], Tomohiro Morohoshi[3], Mitsuaki Ono[4] and Mamoru Satou[1]

Author affiliations:

[1] Research Center of Genetic Resources, National Agriculture and Food Research Organization (NARO), 2-1-2 Kannondai, Tsukuba, Ibaraki 305-8602, Japan

[2] Institute for Plant Protection, NARO, 2-1-18 Kannondai, Tsukuba, Ibaraki 305-8666, Japan

[3] Graduate School of Regional Development and Creativity, Utsunomiya University, 7-1-2 Yoto, Utsunomiya, Tochigi 321-8585, Japan

[4] Yamanashi Agritechnology Center (retired), 1100 Shimoimai, Kai, Yamanashi 400-0105, Japan

*** Correspondence:**

Hiroyuki Sawada, sawada@naro.affrc.go.jp

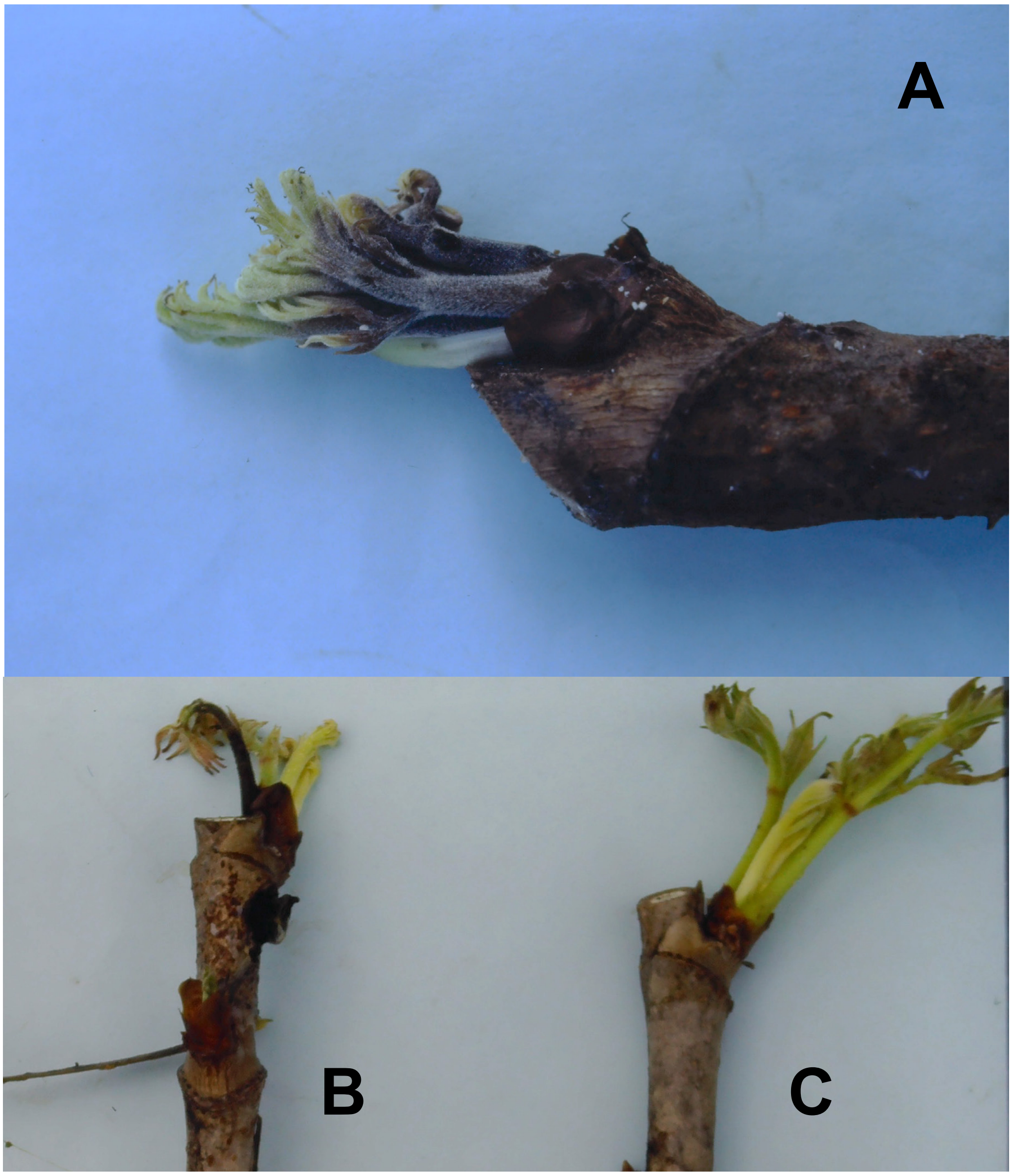

Fig. S1. Symptoms in new shoots of Japanese angelica tree (*Aralia elata*).

Disease symptoms in natural infection in Yamanashi Prefecture, Japan (A). In artificial inoculation tests [11], *Pectobacterium araliae* MAFF 302110^T formed soft rot symptoms (B). Sterile distilled water (negative control) (C).

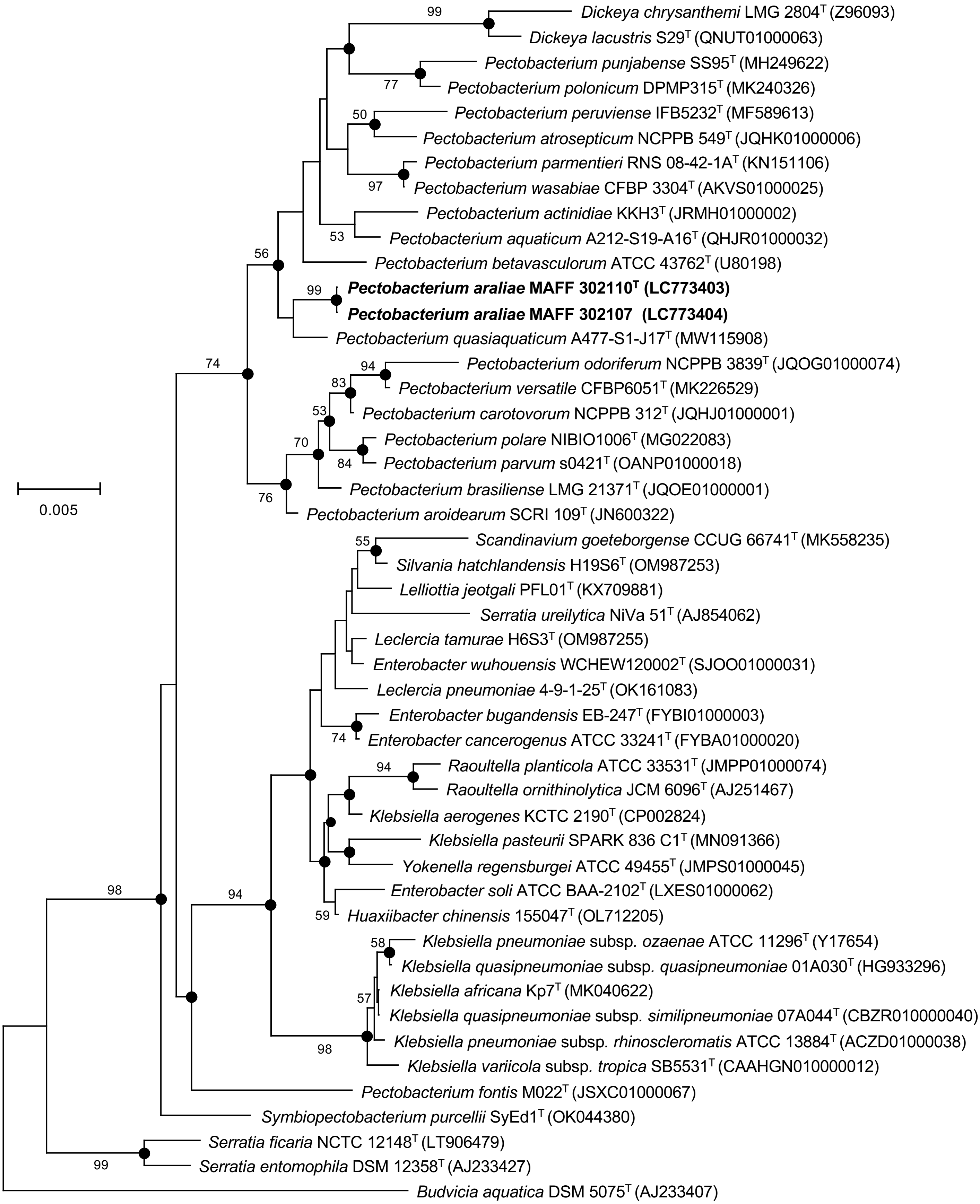

Fig. S2. Neighbour-joining tree based on the 16S rRNA gene sequences, showing the relationships between *Pectobacterium araliae* sp. nov. strains (boldface type) and closely related species (Table S4). *Budvicia aquatica* DSM 5075^{T} was used as an outgroup. Evolutionary distances were computed using the maximum composite likelihood method. Rate variation among sites was modelled with a gamma distribution (shape parameter = 1). T, type strain of the species. Numbers at nodes indicate standard bootstrap values (≥ 50%) from 1000 repetitions. Filled circles indicate that the corresponding nodes were also recovered in the maximum-likelihood and maximum-parsimony trees. Bar, 0.005 substitutions per nucleotide position.

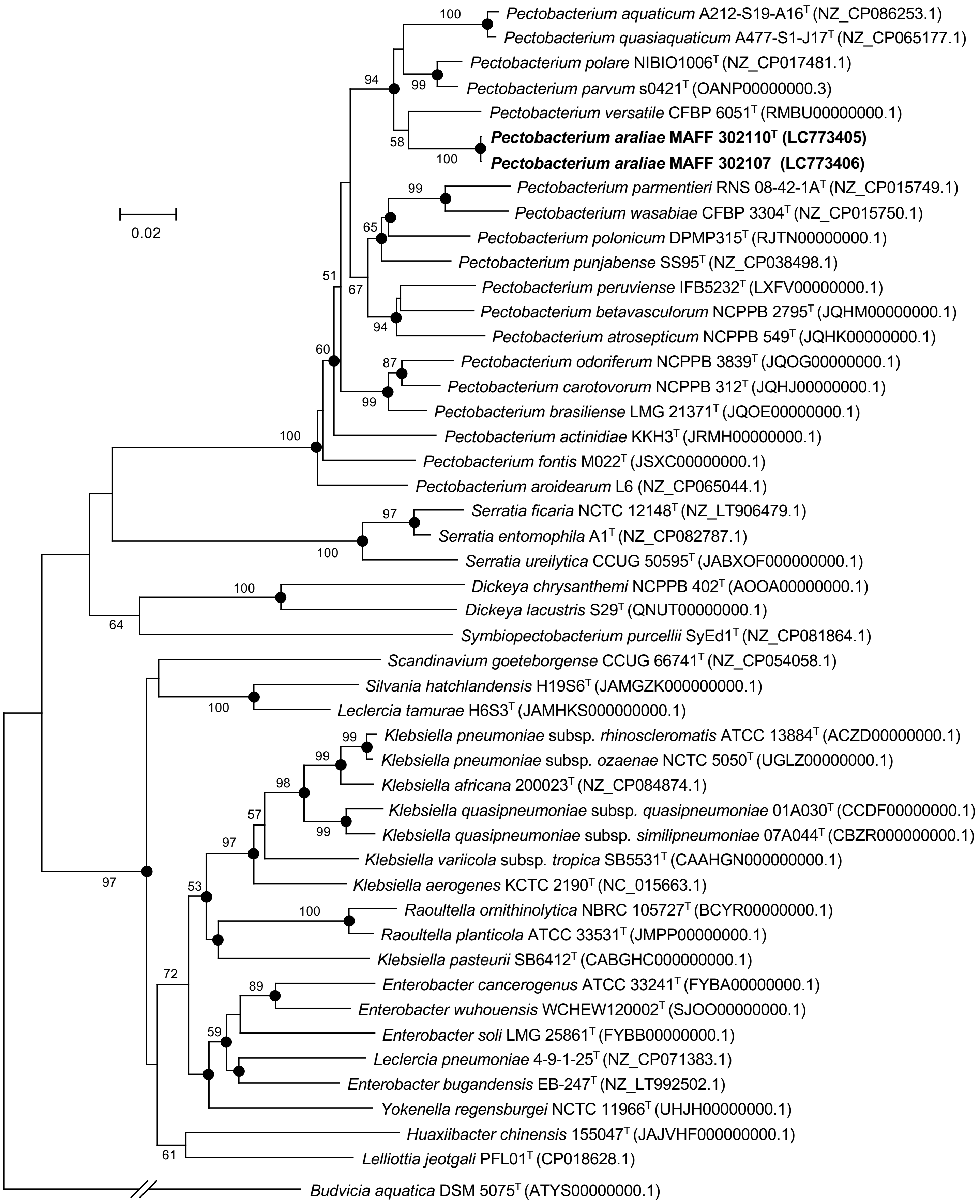

Fig. S3. Neighbour-joining tree based on the *gyrB* gene sequences, showing the relationships between *Pectobacterium araliae* sp. nov. strains (boldface type) and closely related species (Table S4). *Budvicia aquatica* DSM 5075^{T} was used as an outgroup. The sequences used here were extracted from the respective whole genome sequences (accession numbers are in parentheses), except for those of *P. araliae* sp. nov. strains determined in this study. Evolutionary distances were computed using the maximum composite likelihood method. Rate variation among sites was modelled with a gamma distribution (shape parameter = 1). T, type strain of the species. Numbers at nodes are standard bootstrap values (≥ 50%) from 1000 repetitions. Filled circles indicate that the corresponding nodes were also recovered in the maximum-likelihood and maximum-parsimony trees. Bar, 0.02 substitutions per nucleotide position.

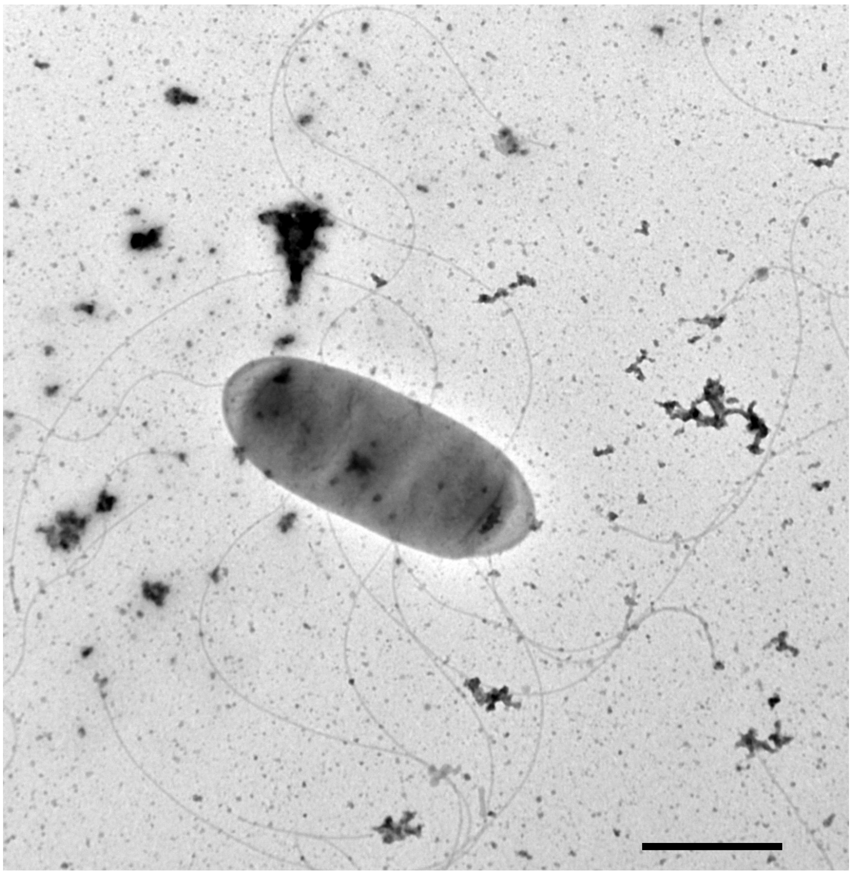

Fig. S4. Transmission electron micrograph of *Pectobacterium araliae* sp. nov. strain MAFF 302110^T, showing a rod-shaped cell and peritrichous flagella. Scale bar, 1 µm.

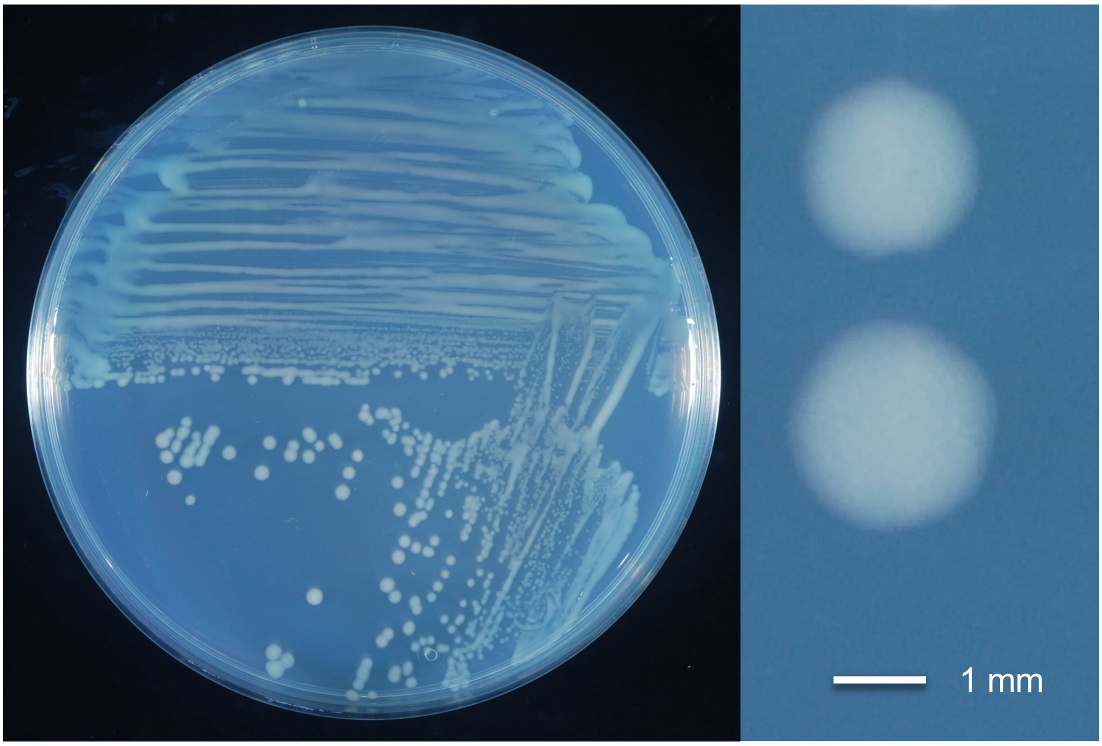

Fig. S5. Colony colour and morphology of *Pectobacterium araliae* sp. nov. strain MAFF 302110^{T} on a TY (tryptone yeast-extract) agar plate incubated at 28 °C for 48 h.

Table S1. Characteristics of the genome sequences

Characteristic		MAFF 302110^T	MAFF 302107
**DDBJ/ENA/GenBank accession number**		AP028908	BRCR00000000
***De novo* assembly**	Genome size (bp)	4,663,678	4,647,160
	G+C content (mol%)	51.1	50.9
	Number of contigs	1	136
	N_{50} contig size (bp)	4,663,678	158,431
	Largest contig size (bp)	4,663,678	300,413
	Average contig size (bp)	4,663,678	34,170
	Coverage	53x	337x
**Completeness check using CheckM ***	Completeness (%)	94.19	94.17
	Contamination (%)	2.09	2.32

* The quality of the assembled genomes was assessed using CheckM [Parks et al., 2015].

Parks DH, Imelfort M, Skennerton CT, Hugenholtz P, Tyson GW. CheckM: assessing the quality of microbial genomes recovered from isolates, single cells, and metagenomes. *Genome Res.* 2015;25:1043–1055.

Table S2. Results of pairwise comparisons of the MAFF 302110^T genome against the type strain genomes performed in TYGS

TYGS (Type Strain Genome Server) [23] is available at https://tygs.dsmz.de. The data shown here were calculated with Formula d_4 (d_4 is identical to GGDC Formula 2) using the MAFF 302110^T genome sequence as a query and have been presented in descending order of their dDDH values.

Species	Strain	dDDH (d_4, in %)	Confidence intervals (d_4, in %)
Pectobacterium polonicum	DPMP315^T	47.1	[44.6 - 49.7]
Pectobacterium punjabense	SS95^T	43.7	[41.2 - 46.2]
Pectobacterium carotovorum	NCPPB 312^T	40.1	[37.6 - 42.6]
Pectobacterium parmentieri	RNS 08-42-1A^T	40.0	[37.5 - 42.5]
Pectobacterium carotovorum	DSM 30168^T	40.0	[37.5 - 42.5]
Pectobacterium wasabiae	CFBP 3304^T	40.0	[37.5 - 42.5]
Pectobacterium carotovorum	ICMP 5702^T	40.0	[37.5 - 42.5]
Pectobacterium versatile	CFBP 6051^T	39.9	[37.4 - 42.5]
Pectobacterium odoriferum	NCPPB 3839^T	39.8	[37.3 - 42.3]
Pectobacterium aquaticum	A212-S19-A16^T	38.6	[36.2 - 41.2]
Pectobacterium polare	NIBIO1006^T	38.4	[35.9 - 40.9]
Pectobacterium actinidiae	KKH3^T	38.3	[35.9 - 40.8]
Pectobacterium quasiaquaticum	A477- S1- J17^T	37.6	[35.1 - 40.1]
Pectobacterium brasiliense	LMG 21371^T	37.4	[34.9 - 39.9]
Pectobacterium betavasculorum	NCPPB 2795^T	36.2	[33.7 - 38.7]
Serratia ficaria	NBRC 102596^T	20.5	[18.3 - 22.9]

Table S3. Results of pairwise comparisons of the MAFF 302110^T genome against the type strain genomes performed in Taxonomy Check implemented in DFAST

DFAST (DDBJ Fast Annotation and Submission Tool) [16] is available at https://dfast.ddbj.nig.ac.jp. The data shown here were calculated wiht the FastANI algorithm [24] using the MAFF 302110^T genome sequence as a query and have been presented in descending order of their ANI values.

Species	Strain	Accession	Taxonomy ID	ANI (%)	Matched fragments	Total fragments	Status
Pectobacterium polonicum	DPMP315^T	GCA_005497185.1	2485124	92.224	1250	1554	below_threshold
Pectobacterium punjabense	SS95^T	GCA_012427845.1	2108399	91.115	1237	1554	below_threshold
Pectobacterium punjabense	SS95^T	GCA_003028395.1	2108399	91.068	1201	1554	below_threshold
Pectobacterium versatile	CFBP 6051^T	GCA_004296685.1	2488639	90.543	1245	1554	below_threshold
Pectobacterium carotovorum subsp. *carotovorum*	ICMP 5702^T	GCA_001039055.1	555	90.490	1241	1554	below_threshold
Pectobacterium carotovorum	DSM 30168^T	GCA_900129615.1	554	90.472	1247	1554	below_threshold
Pectobacterium carotovorum subsp. *carotovorum*	NCPPB 312^T	GCA_000749855.1	555	90.463	1240	1554	below_threshold
Pectobacterium odoriferum	NCPPB 3839^T	GCA_000754765.1	78398	90.287	1208	1554	below_threshold
Pectobacterium wasabiae	CFBP 3304^T	GCA_000291725.1	55208	89.912	1232	1554	below_threshold
Pectobacterium wasabiae	CFBP 3304T	GCA_001742185.1	55208	89.869	1252	1554	below_threshold
Pectobacterium parmentieri	RNS 08-42-1A^T	GCA_001742145.1	1905730	89.864	1252	1554	below_threshold
Pectobacterium polaris	NIBIO1006^T	GCA_002307355.1	2042057	89.788	1230	1554	below_threshold
Pectobacterium aquaticum	A212-S19-A16^T	GCA_003382565.3	2204145	89.668	1133	1554	below_threshold
Pectobacterium parvum	s0421^T	GCA_900195285.2	2778550	89.636	1173	1554	below_threshold
Pectobacterium brasiliense	CFBP 6617^T	GCA_016950255.1	180957	89.408	1256	1554	below_threshold
Pectobacterium brasiliense	LMG 21371^T	GCA_000754695.1	180957	89.361	1234	1554	below_threshold
Pectobacterium quasiaquaticum	A477-S1-J17^T	GCA_014946775.2	2774015	89.175	1172	1554	below_threshold
Pectobacterium betavasculorum	NCPPB 2795^T	GCA_000749845.1	55207	88.644	1177	1554	below_threshold
Pectobacterium colocasium	LJ1^T	GCA_020181655.1	2878098	88.625	1217	1554	below_threshold
Serratia entomophila	A1^T	GCA_021462285.1	42906	78.901	380	1554	below_threshold
Serratia bockelmannii	S3^T	GCA_008011855.1	2703793	78.835	386	1554	below_threshold
Serratia nevei	S15^T	GCA_008364245.1	2703794	78.528	389	1554	below_threshold

Table S4. 16S rRNA gene sequence similarities between *Pectobacterium araliae* sp. nov. strain MAFF 302110^T and type strains of closely related species

Similarity values based on the 16S rRNA gene sequences were calculated with the pairwise nucleotide sequence alignment tool in the EzBioCloud [27] using the MAFF 302110^T sequence as a query. The data shown here have been presented in descending order of the similarity values calculated.

Species	Strain	Accession number *	Pairwise Similarity (%)
Pectobacterium quasiaquaticum	A477-S1-J17^T	MW115908	99.36
Pectobacterium aquaticum	A212-S19-A16^T	QHJR01000032	99.15
Pectobacterium aroidearum	SCRI 109^T	JN600322	98.94
Pectobacterium polonicum	DPMP315^T	MK240326	98.85
Pectobacterium actinidiae	KKH3^T	JRMH01000002	98.79
Pectobacterium carotovorum	NCPPB 312^T	JQHJ01000001	98.72
Pectobacterium wasabiae	CFBP 3304^T	AKVS01000025	98.65
Pectobacterium brasiliense	LMG 21371^T	JQOE01000001	98.58
Pectobacterium parmentieri	RNS 08-42-1A^T	KN151106	98.51
Pectobacterium polare	NIBIO1006^T	MG022083	98.30
Pectobacterium versatile	CFBP6051^T	MK226529	98.30
Pectobacterium parvum	s0421^T	OANP01000018	98.23
Pectobacterium betavasculorum	ATCC 43762^T	U80198	98.23
Pectobacterium fontis	M022^T	JSXC01000067	98.16
Pectobacterium atrosepticum	NCPPB 549^T	JQHK01000006	98.09
Pectobacterium punjabense	SS95^T	MH249622	98.09
Scandinavium goeteborgense	CCUG 66741^T	MK558235	98.01
Symbiopectobacterium purcellii	SyEd1^T	OK044380	97.94
Dickeya lacustris	S29^T	QNUT01000063	97.87
Raoultella ornithinolytica	JCM 6096^T	AJ251467	97.79
Pectobacterium odoriferum	NCPPB 3839^T	JQOG01000074	97.73
Klebsiella quasipneumoniae subsp. *similipneumoniae*	07A044^T	CBZR010000040	97.73
Klebsiella aerogenes	KCTC 2190^T	CP002824	97.73
Klebsiella variicola subsp. *tropica*	SB5531^T	CAAHGN010000012	97.73
Klebsiella pneumoniae subsp. *rhinoscleromatis*	ATCC 13884^T	ACZD01000038	97.66
Enterobacter wuhouensis	WCHEW120002^T	SJOO01000031	97.66
Leclercia pneumoniae	4-9-1-25^T	OK161083	97.65
Klebsiella quasipneumoniae subsp. *quasipneumoniae*	01A030^T	HG933296	97.65
Pectobacterium peruviense	IFB5232^T	MF589613	97.63
Silvania hatchlandensis	H19S6^T	OM987253	97.62
Enterobacter cancerogenus	ATCC 33241^T	FYBA01000020	97.59
Leclercia tamurae	H6S3^T	OM987255	97.54
Serratia ureilytica	NiVa 51^T	AJ854062	97.52
Klebsiella africana	Kp7^T	MK040622	97.52
Klebsiella pasteurii	SPARK_836_C1^T	MN091366	97.52
Huaxiibacter chinensis	155047^T	OL712205	97.52
Dickeya chrysanthemi	LMG 2804^T	Z96093	97.51
Klebsiella pneumoniae subsp. *ozaenae*	ATCC 11296^T	Y17654	97.50
Lelliottia jeotgali	PFL01^T	KX709881	97.46
Yokenella regensburgei	ATCC 49455^T	JMPS01000045	97.44
Raoultella planticola	ATCC 33531^T	JMPP01000074	97.44
Enterobacter soli	ATCC BAA-2102^T	LXES01000062	97.44
Enterobacter bugandensis	EB-247^T	FYBI01000003	97.44
Serratia ficaria	NCTC 12148^T	LT906479	97.30
Serratia entomophila	DSM 12358^T	AJ233427	97.02

* The 16S rRNA gene sequences used here are the same as those used in the phylogenetic analyses (Fig. S2).

Table S5. Number of genes in the MAFF 302110^T genome assigned to each KEGG-defined function by using the BlastKOALA tool *

Category/Subcategory	Gene counts
**Metabolism**	
Carbohydrate metabolism	338
Energy metabolism	157
Lipid metabolism	61
Nucleotide metabolism	92
Amino acid metabolism	225
Metabolism of other amino acids	60
Glycan biosynthesis and metabolism	79
Metabolism of cofactors and vitamins	176
Metabolism of terpenoids and polyketides	33
Biosynthesis of other secondary metabolites	57
Xenobiotics biodegradation and metabolism	45
**Genetic Information Processing**	
Transcription	4
Translation	78
Folding, sorting and degradation	54
Replication and repair	82
Information processing in viruses	1
**Environmental Information Processing**	
Membrane transport	251
Signal transduction	131
**Cellular Processes**	
Transport and catabolism	5
Cell growth and death	19
Cellular community - prokaryotes	126
Cell motility	63
**Organismal Systems**	
Immune system	6
Endocrine system	17
Digestive system	1
Nervous system	2
Development and regeneration	1
Aging	8
Environmental adaptation	7
**Human Diseases**	
Cancer: overview	18
Cancer: specific types	5
Infectious disease: viral	1
Infectious disease: bacterial	24
Infectious disease: parasitic	3
Immune disease	2
Neurodegenerative disease	6
Cardiovascular disease	12
Endocrine and metabolic disease	6
Drug resistance: antimicrobial	49
Drug resistance: antineoplastic	6

* BlastKOALA [42] is available at https://www.kegg.jp/blastkoala/.

Table S6. Number of domains in the genomes of MAFF 302110^T and its closely related *Pectobacterium* species, assigned to each CAZyme (carbohydrate-active enzyme) class by using the dbCAN3 meta-server *

Strains: 1, *Pectobacterium araliae* MAFF 302110^T; 2, *Pectobacterium polonicum* DPMP315^T; 3, *Pectobacterium parmentieri* RNS 08-42-1A^T; 4, *Pectobacterium punjabense* SS95^T; 5, *Pectobacterium wasabiae* CFBP 3304^T.

CAZyme class	Number of domains assigned to each CAZyme class †				
	1	2	3	4	5
Auxiliary activities (AAs)	2	2	2	2	1
Carbohydrate-binding modules (CBMs)	11	14	14	15	12
Carbohydrate esterases (CEs)	8	8	9	8	9
Glycoside hydrolases (GHs)	47	53	52	50	46
Glycosyl transferases (GTs)	30	28	35	31	35
Polysaccharide lyases (PLs)	13	17	14	16	14
Total	111	122	126	122	117

* dbCAN3 meta-server [49] is available at https://bcb.unl.edu/dbCAN2/index.php.

† If an annotation was supported by at least two of the following tools/databases in dbCAN3: DIAMOND (CAZy), HMMER (dbCAN), or HMMER (dbCAN-sub), the hit was assigned to the corresponding CAZyme class. The total number of domains, assinged to each CAZyme class, is shown for each strain in this table.

Table S7. Number of genes in the genomes of MAFF 302110^T and its closely related *Pectobacterium* species, assigned to each secretion system by using the BlastKOALA tool *

Strains: 1, *Pectobacterium araliae* MAFF 302110^T; 2, *Pectobacterium polonicum* DPMP315^T; 3, *Pectobacterium parmentieri* RNS 08-42-1A^T; 4, *Pectobacterium punjabense* SS95^T; 5, *Pectobacterium wasabiae* CFBP 3304^T.

**Secretion system**	**Number of genes assigned to each secretion system †**				
	**1**	**2**	**3**	**4**	**5**
Sec Secretory Pathway	12	12	12	12	12
Tat Secretory Pathway	4	5	4	4	4
Type II Secretion System	13	13	13	13	13
**Type III Secretion System ‡**	0	**11**	0	**11**	0
**Type IV Secretion System**	**9**	0	**9**	0	**9**
**Type VI Secretion System**	**20**	**10**	**22**	0	**23**
Total	58	51	60	40	61

* BlastKOALA [42] is available at https://www.kegg.jp/blastkoala/.

† The total number of genes, assinged to the core components of each secretion system by using the BlastKOALA, is shown for each strain.

‡ Excluding the flagellar secretion system.
